# Liver regeneration in traditional Chinese medicine: advances and challenges

**DOI:** 10.1051/rmr/190003

**Published:** 2020-01-16

**Authors:** Feng Zhang, Feixia Wang, Baoyu Liang, Zhanghao Li, Jiangjuan Shao, Zili Zhang, Shijun Wang, Shizhong Zheng

**Affiliations:** 1 Jiangsu Key Laboratory for Pharmacology and Safety Evaluation of Chinese Materia Medica, School of Pharmacy, Nanjing University of Chinese Medicine Nanjing 210023 PR China; 2 Jiangsu Key Laboratory of Therapeutic Material of Chinese Medicine, School of Pharmacy, Nanjing University of Chinese Medicine Nanjing 210023 PR China; 3 State Key Laboratory Cultivation Base for TCM Quality and Efficacy, School of Pharmacy, Nanjing University of Chinese Medicine Nanjing 210023 PR China; 4 Shandong Co-innovation Center of TCM Formula, College of Traditional Chinese Medicine, Shandong University of Traditional Chinese Medicine Jinan 250355 PR China

**Keywords:** Liver regeneration, traditional Chinese medicine, hepatocyte, liver injury, imbalance, marrow

## Abstract

Liver diseases pose a serious problem for national health care system all over the world. Liver regeneration has profound impacts on the occurrence and development of various liver diseases, and it remains an extensively studied topic. Although current knowledge has suggested two major mechanisms for liver regeneration, including compensatory hyperplasia of hepatocytes and stem or progenitor cell-mediated regeneration, the complexity of this physiopathological process determines that its effective regulation cannot be achieved by single-target or single-component approaches. Alternatively, using traditional Chinese medicine (TCM) to regulate liver regeneration is an important strategy for prevention and treatment of liver disorder and the related diseases. From the perspectives of TCM, liver regeneration can be caused by the disrupted balance between hepatic damage and regenerative capacity, and the “marrow”-based approaches have important therapeutic implications for liver regeneration. These two points have been massively supported by a number of basic studies and clinical observations during recent decades. TCM has the advantages of overall dynamic fine-tuning and early adjustment, and has exhibited enormous therapeutic benefits for various liver diseases. Here, we review the recent advances in the understanding of liver regeneration in TCM system in the hope of facilitating the application of TCM for liver diseases via regulation of liver regeneration.

## 1 Introduction

It has been one of the directions that academic community strives to prevent and treat liver diseases from the perspective of internal mechanism of human body, and more attention has been gained in recent years. The mechanism of liver regeneration has long-termly been an important host factor for the development of liver diseases. After nearly a century of efforts, especially with the rapid development of stem cell-centered regenerative medicine, mechanistic understanding of liver regeneration progresses rapidly [1]. However, studies with high level evidence-based measures and methods for regulating liver regeneration are still relatively limited.

It has currently been known that the regulation of liver regeneration is not only the partial function of the liver, but also the overall comprehensive effect of dynamic changes of human body. Therefore, regulation of liver regeneration must be based on the overall dynamic regulation. The treatment using one-sided therapeutic method will fail to meet the needs of various aspects and ever-changing regulation of liver regeneration, and the curative effect must be limited [2,3]. Due to the functional characteristics of multi-target, multi-level and multi-system of the overall dynamic fine-tuning adjustment, traditional Chinese medicine (TCM) has gradually been a research hotspot, especially given that it can meet the needs of regulating liver regeneration in multi-aspects, complexity and fickleness [4]. Here, we present a brief review of the general cellular and molecular mechanisms of liver regeneration followed by detailed discussion on the recognition of liver regeneration in TCM theoretical system and its therapeutic implications.

## 2 General mechanism of liver regeneration

Current paradigm generally describes two mechanisms of liver regeneration: hepatocyte compensatory hyperplasia, and stem or progenitor cell-driven regeneration [5]. It is acknowledged that hepatic parenchymal turnover is maintained solely through hepatocyte division controlled by some pivotal signaling pathways, including Wnt/β-catenin and Hippo/Yap [6,7]. This spatiotemporal regulation contributes to metabolic zonation and determines hepatocyte function [8]. Rodent models such as the classical partial hepatectomy in rats demonstrate that the liver size is restored through hepatocyte hypertrophy and hyperplasia in the remaining lobes. This is a highly regulated process dependent on changes in blood flow, sinusoidal endothelium, immune cells, hepatic stellate cells, and a host of growth factors and paracrine signals [9,10]. This regenerative potential is both highly efficient and of almost infinite capacity.

For the alternative regeneration mechanism, blood-borne cells of the bone marrow origin also play a role, in addition to the resident liver cells [11–13]. Hepatic stellate cells (HSCs) are also an important element of the liver regeneration machinery being part of liver stem cell niche, supporting regeneration at early stages by producing growth factors and inducing regeneration arrest after restoration of normal organ mass [14]. Notably, recent studies have concentrated on determining the contribution of expansion of the putative hepatic progenitor cell population resident in the bile ductules. It appears that when the ability of hepatocytes to proliferate is inhibited or overwhelmed, the liver maintains the ability to regenerate via mobilization of a population of hepatic progenitor cells [15,16]. For example, in the zebrafish models, where hepatocytes are ablated or prevented from entering the cell cycle, hepatic progenitor cells are activated, proliferate and can restore the hepatocyte mass ensuring survival of the organism [17]. However, much debate has surrounded the true significance of this hepatic progenitor cell pool, awaiting further elucidation.

## 3 Understanding of liver regeneration from perspectives of TCM

### 3.1 Imbalance between liver injury and regeneration

Having experienced the research hotspots, including anti-virus, anti-liver fibrosis, anti-hepatoma and immune-regulation for the treatment of liver diseases, a new research hotspot of regulating liver regeneration by TCM has been emerged. Many past studies have paid attention to studying the effects of TCM on liver damage or liver regeneration and to elucidating the underlying mechanisms [18]. With the deepening of research, the biological nature of “deficiency syndrome” has been demonstrated to be the imbalance between pathological damage and tissue regeneration. In recent years, the regulatory mechanisms for TCM coordination of liver damage and liver regeneration for treatment of liver diseases have gradually been acknowledged [19]. Through understanding the nature of “hepatic deficiency syndrome” from the perspective of TCM vitality theory, the progression of liver disease syndrome can be roughly divided into several development trends or outcome patterns as follows. (1) The damaged liver structure can be repaired by induced regeneration in a short and mild liver injury. The liver damage and liver regeneration are only temporarily imbalanced, and the equilibrium state is quickly restored. In this case, liver physiological function is not significantly affected, and the liver disease-related deficiency syndrome is not formed, and the disease syndrome tends to recover. Most self-healing liver diseases follow such trend or outcome pattern. (2) The liver damage is too severe and/or too urgent, which is beyond the repair capacity of liver regeneration. The patients are too late and/or insufficient to regenerate the heavy liver damage, in which the liver damage and liver regeneration are seriously imbalanced, and the patients will be dead because of acute failure of liver function. Patients with acute liver failure who are too late for emergent treatment follow this trend or outcome pattern. (3) Although liver injury is not urgent or heavy, it will be repeated or persistent, eventually leading to disorder of liver regeneration and repair. Under such a condition, the liver damage and regeneration will be imbalanced, affecting liver tissue remodeling and functional recovery, and forming liver disease-related “deficiency syndrome”. The progressive and incurable chronic liver disease syndromes follow this trend or outcome pattern. Taken together, it can be concluded that one of the basic rules for prevention and treatment of liver disease syndrome is to regulate the imbalance between liver damage and liver regeneration, and that is, to reduce liver damage concomitant with promoting the normal repair mechanism of liver regeneration. Under normal circumstances, reducing liver damage is conducive to liver regeneration and repair, and regulating liver regeneration and repair is also beneficial for alleviating liver damage. Both aspects are mutually beneficial for and complement to each other, which is a virtuous circulation. Thus, the correction of the imbalance between liver damage and regeneration will be the most efficient, and the treatment effect will be the best.

The therapeutic practice of TCM or integrated Chinese and Western medicine for hepatic failure has demonstrated the scientific and advanced nature of reducing the mortality of liver failure, alleviating complications, and improving the quality of life by regulating the imbalance between liver damage and liver regeneration [20–27]. TCM believes that the damp, heat, and epidemic pathogenic factors are the main cause of hepatic failure. The pathological changes caused by damp, heat and epidemic pathogenic factors are mainly inflammatory injuries and necrosis. The Chinese medicines for clearing heat and promoting diuresis and detoxification exhibit the dual effects of anti-inflammation and regulation of liver regeneration. Such medicines used most commonly include rheum officinale and its compound preparations, capillary artemisia and its compound preparations, liquorice and its modern preparations (glycyrrhizic acid glycosides, diammonium glycyrrhizinate oral and intravenous preparations), etc. Animal studies and clinical trials have confirmed that rheum officinale can alleviate hepatocyte damages induced by inflammatory factors such as TNF-α, IL-1 and IL-6 due to enteral endotoxemia, promoting liver regeneration [28,29]. The water-soluble active ingredients of capillary artemisia can reduce the activities of ALT and AST, and increase the activity of superoxide dismutase to produce anti-oxidative effects in animal models of liver injury. IL-6 and TNF-α are essential regulatory factors for initiating liver regeneration, but if overexpressed, they become inflammatory mediators leading to liver damage. Capillary artemisia can inhibit the overexpression of inflammatory mediators such as IL-6 and TNF-α in liver tissues, so as to produce anti-inflammatory and liver protective effects. On the other hand, capillary artemisia can also promote liver protein synthesis and liver regeneration, and accelerate the repair of necrotic liver tissues by increasing ribonucleic acids and hepatic glycogen [30]. Capillary mixture can reduce the expression of TNF-α, promote the expression of hepatocyte growth factor, and inhibit the apoptosis of hepatocytes in model rats with fulminant hepatic failure [31]. The ethanolic extracts of capillary artemisia have direct and significant protective effects on FFA-caused hepatotoxicity, and the underlying mechanism may be related to inhibition of excessive activation of nuclear factor-κB [32,33]. The effective constituents glycyrrhizic acid and glycyrrhetinic acid in licorice can alleviate liver degeneration and necrosis caused by drug-induced liver damage in animals, exerting significant anti-inflammatory and hepatoprotective effects via regulating the multi-stages of inflammatory progression [34]. Furthermore, liver sinusoidal endothelial cells play a very important role in the occurrence and development of liver injury. Shen et al. found that isoliquiritigenin and diamine glycyrrhizate have significant protective effects against CoCl_2_-induced hypoxia injuries in SK-HEP-1 cells, and meanwhile significantly improved the NO content and NOS activity concomitant with a decrease in reactive oxygen content [35]. Studies have shown that the expression of NF-κB in patients with severe hepatitis and liver failure is significantly lower than that in the patients with normal or mild liver injury [36]. Specific enhancement of NF-κB expression in hepatocytes can reduce liver damage and promote hepatocyte regeneration and repair [37]. In addition, Qiu et al. found that the compound glycyrrhizin can reduce the severity of acute liver injury in mice by regulating NF-κB pathway [38].

### 3.2 Liver regeneration via “marrow”

“Marrow engenders liver” is an important academic thought of *Nei Jing* [39,40]. Recent studies have revealed that the biological nature of “marrow” is the stem cells and related tissue microenvironment [41]. “Marrow engenders liver” includes at least two mechanisms for liver repair and regeneration: direct transformation of “marrow” into liver, and regulatory transformation of “marrow” into liver. Through the central link of the “marrow”, TCM can make the pathophysiological changes of “marrow” conform to and meet the needs of occurrence and development of regenerative repair, so as to maintain or promote the physiological state of “liver generation via marrow”, and to prevent or improve the pathological state of hypofunction of “liver generation via marrow”. In short, “engendering marrow” is to make marrow engendered, which is to maintain or promote the normal physiological state of “marrow” [42]. Therefore, comprehensive use of the following two strategies shall be adopted: on the one hand, to maintain or promote the normal physiological state of hepatic stem cells and their tissue microenvironment; on the other hand, to prevent or improve the abnormal pathological state of hepatic stem cells and their tissue microenvironment. The two strategies complement to each other.

Recently, some studies have found that the increasingly severe liver cirrhosis causes hematopoietic dysfunction and loss of hematopoietic stem cells, leading to dysfunction of blood and immunity and reduced regeneration potential [43]. Thus, the idea that restoring bone marrow function may provide new treatment option for cirrhosis has been proposed. Based on the “marrow”-centered therapeutic targets, TCM has established the treatment principle of “reinforcing kidney, engendering marrow, and generating liver”, which effectively guides the prevention and treatment of liver diseases and the related disorders. During the past 20 yr, a number of studies with in-depth experiments and clinical research around this treatment principle have been carried out [44–55]. The results show that this treatment principle is able to delay, prevent, or even reverse the occurrence and development of chronic liver disease, liver failure, cirrhosis, and liver cancer, and relatively high level evidence-based medical evidence has been obtained. The therapeutic mechanisms of Zuogui Pill, Diwu Yanggan Capsule, Kangdu Ruanjian Capsule and Jianghuang Capsule, which reflect the treatment principle of “reinforcing kidney, engendering marrow, and generating liver”, involve multiple pathways and links such as hypothalamic-pituitary-hepatic axis, neuro-endocrine-immuno-network of regulation of liver regeneration, transformation of bone marrow stem cells into liver cells, and liver tissue microenvironment, etc. [44–55]. By using cross-sex bone marrow transplantation model, multiple liver injury animal models, MSG-rat-liver regeneration model, bone marrow stem cell and hepatocyte co-culture technology, gene chip technology, protein mass spectrometry, multiple key proteins and their interaction mechanisms involved in “reinforcing kidney, engendering marrow, and generating liver” affecting the transformation of bone marrow stem cells into hepatocytes have been uncovered [44–55]. Multiple signal pathways related to this principle and to the regulation of liver regeneration have been found, such as Wnt, mitogen-activated protein kinase, TGF-β, Jak-STAT, Toll-like receptors, etc. [44–55].

## 4 Therapeutic regulation of liver regeneration by TCM

Because of the extremely complex and verified nature of the process and mechanism of regeneration and repair, in order to maintain or promote the regeneration of the stem cell microenvironment, it is necessary to use the mode of action of overall dynamic fine-tuning early adjustment to comprehensively regulate with multiple targets and multiple pathways. Obviously, single-target chemical medicines cannot meet such requirements. Therefore, there is currently no single-target chemical medicine that can regulate liver regenerative repair effectively. TCM has the comprehensive adjustment advantages of complex composition and overall dynamic fine-tuning features, which fully meet the needs of regulating regeneration and restoration. TCM utilizes the natural healing ability of viscera tissue to regenerate and repair the damage of viscera tissue, and rebuild and restore the structure and function of visceral tissue, which possesses the characteristics of being natural, making the best use of circumstances, dynamic changes, reversing the disease, overall adjustment, high security, and being affirmative in maintaining or improving the regenerative repaired stem cell microenvironment [56]. It has been known that IL-6 and TNF-α are key hepatic inflammation-inducing factors and their overexpression induces hepatic inflammatory damage, which is positively correlated with the severity of liver disease [57]. It has been found that TCM is able to alleviate inflammatory damage in the liver by inhibiting the overexpression of IL-6 and TNF-α [58–60].

Proliferation and activation of HSCs are thought to be the key steps of liver fibrosis, and the attention of treatment has always been focused on how to inhibit the proliferation and activation of HSCs [61]. However, as the research progresses, it is recognized that HSCs also play an important role in liver regeneration as a component of stem cells [62]. In recent years, it has been confirmed that HSCs have the potential of multi-directional differentiation, which can be differentiated into hepatocytes and vascular endothelial cells under certain conditions, and can directly participate in cell regeneration of liver injury repair [63]. Considering the impact on HSCs from the perspective of regulating liver regeneration, it is not necessary to blindly emphasize the inhibition of HSC proliferation and activation, but more attention shall be focused on how to regulate the differentiation direction of HSCs, and to promote the coordinated development of liver regeneration. TGF-β1, epithelial-mesenchymal transition, hedgehog signaling pathways are important mechanisms affecting HSC proliferation and differentiation [64]. Recent studies found that the TCM compound Diwu Yanggan Capsule is able to inhibit the overexpression of TGF-β1, inhibit the overactivation of hedgehog signaling pathway, prevent the occurrence and development of hepatic fibrosis, and promote liver regeneration and repair by inhibiting the EMT process of HSCs and/or promoting its MET process [65–67].

Current studies have confirmed that the oval cells are one kind of important hepatic stem cells for liver regeneration and repair [68,69]. When hepatocyte proliferation is inhibited or hepatocyte regeneration is insufficient to meet the regenerative repair, oval cell proliferation becomes an important way for liver regeneration and repair. However, oval cells are also progenitor cells of liver cancer, and their long-term excessive hyper-proliferation may promote the occurrence and development of liver cancer [70]. Studies have found that the TCM compound preparation Diwu Yanggan Capsule is able to promote the proliferation and differentiation of bone marrow stem cells and intrahepatic oval cells in 2-AAF/PH model rats at the early and middle stages, which is conducive to liver regeneration and repair [71]. It is also able to inhibit the excessive proliferation and abnormal differentiation of liver oval cells at the middle and late stages, which is conducive to prevent and treat hepatocellular carcinogenesis. Disruption of Wnt/β-catenin pathway by Diwu Yanggan Capsule may be the molecular mechanism of inhibiting excessive proliferation and abnormal differentiation of oval cells [71]. Regulating the expression of multiple liver regeneration-related cytokines (TNF-α, IL-1, vascular endothelial growth factor, and interferon-γ) in 2-AAF/PH rat models makes them more inclined to the normal levels, thereby inhibiting the excessive proliferation and abnormal differentiation of oval cells and preventing the occurrence and development of precancerous lesions by improving the liver regeneration microenvironment [71]. Moreover, the Solt-Farbe two-step method can be adopted to replicate the liver cancer model in rats, in which the liver regeneration microenvironment contains inhibited hepatocyte regeneration and hyperproliferative oval cells. In this model, the pathogenesis of transforming bone marrow stem cells into liver cancer stem cells exists in liver cancer, and Diwu Yanggan Capsule inhibits the occurrence and development of liver cancer. Its mechanism may be to inhibit the transformation of bone marrow stem cells into liver cancer stem cells, promote the regeneration and repair of hepatocytes, inhibit the excessive proliferation of oval cells, regulate the imbalance of EMT/MET (inhibition of EMT and promote MET), affect the expression of proteins in JAK/STAT and Ras/Raf/Mek/Erk signaling pathways, and improve the liver regeneration microenvironment [72–74].

## 5 Conclusions and challenges

The rapid progress of liver regeneration research, the lack of specific technologies and methods for regulating liver regeneration, and the effective functional characteristics and advantages that the TCM has to regulate liver regeneration, have collectively brought great opportunities for the basic and clinical researches of preventing and curing liver diseases and related diseases by regulating liver regeneration. Many efforts have been made to improve the ability and level of TCM or integrated Chinese and Western medicine to prevent and cure liver diseases and related diseases. However, faced with the more complex interaction mechanism between the components of TCM and the complex mechanism of liver regeneration, and with the insufficiency of clinical standard system for clinical application, insurmountable challenges for basic and clinical application research of regulating liver regeneration with TCM are proposed.

Normal and abnormal liver regeneration are difficult to be distinguished clinically. It is difficult to establish an index system suitable for clinical application to objectively evaluate the normal or abnormal liver regeneration, which is a key scientific problem that hinders the clinical application of TCM to regulate liver regeneration. TCM regulates liver regeneration mainly through inartificial or natural methods to adjust the internal mechanism of liver regeneration and to normalize liver regeneration. The main mechanism of action is to transform the pathological imbalance mechanism into the normal equilibrium mechanism, so the criterion for therapeutic effect evaluation is “expectation of balance”. The complex composition in TCM, the use of natural therapy, the overall dynamic fine-tuning and early adjustment, and the characteristics of individualized diagnosis and treatment exactly meet the complex and ever-changing needs of liver regeneration regulation, which is of realistic and potential advantages. Furthermore, there are needs to investigate how the physical liver regeneration and pathological liver regeneration are regulated by TCM, given that many Chinese herbs medicines have food function and are commonly used in diets of healthy people. In short, the continuous improvement and development of various systems biology technologies can provide ways and hope to solve the above-mentioned complex problems. The combination of evidence-based medical evidence and the real-world big data can also promote clinical research and application of TCM for regulating liver regeneration.

## Conflict of interests

The authors declare that there is no financial or other relationship that might lead to a conflict of the present article. All authors have reviewed the final version of the manuscript and approved it for publication.

## References

[1] Zlotorynski E (2018), Histone methylation boosts liver regeneration. Nat Rev Mol Cell Biol 20, 454–455. 10.1038/s41580-019-0157-8 31273301

[2] Zafarnia S, Mrugalla A, Rix A, Doleschel D, Gremse F, Wolf SD, Buyel JF, Albrecht U, Bode JG, Kiessling F, Lederle W (2019) Non-invasive imaging and modeling of liver regeneration after partial hepatectomy. Front Physiol 10, 904 . 31379606 10.3389/fphys.2019.00904 PMC6652107

[3] Wang S, Zhang C, Hasson D, Desai A, SenBanerjee S, Magnani E, Ukomadu C, Lujambio A, Bernstein E, Sadler KC (2019) Epigenetic compensation promotes liver regeneration. Dev Cell 50, 43–56.e6 . 31231040 10.1016/j.devcel.2019.05.034 PMC6615735

[4] Van Haele M, Snoeck J, Roskams T (2019), Human liver regeneration: an etiology dependent process. Int J Mol Sci 20, pii: E2332 . 31083462 10.3390/ijms20092332 PMC6539121

[5] Valizadeh A, Majidinia M, Samadi-Kafil H, Yousefi M, Yousefi B (2019), The roles of signaling pathways in liver repair and regeneration. J Cell Physiol, DOI: 10.1002/jcp.28336 . 30770551

[6] Li N, Kong M, Zeng S, Hao C, Li M, Li L, Xu Z, Zhu M, Xu Y (2019), Brahma related gene 1 (Brg1) contributes to liver regeneration by epigenetically activating the Wnt/β-catenin pathway in mice. FASEB J 33, 327–338 . 30001167 10.1096/fj.201800197R

[7] Pepe-Mooney BJ, Dill MT, Alemany A, Ordovas-Montanes J, Matsushita Y, Rao A, Sen A, Miyazaki M, Anakk S, Dawson PA, Ono N, Shalek AK, van Oudenaarden A, Camargo FD (2019) Single-cell analysis of the liver epithelium reveals dynamic heterogeneity and an essential role for YAP in homeostasis and regeneration. Cell Stem Cell 25, 23–38.e8 . 31080134 10.1016/j.stem.2019.04.004 PMC6814390

[8] Preziosi M, Okabe H, Poddar M, Singh S, Monga SP (2018) Endothelial Wnts regulate β-catenin signaling in murine liver zonation and regeneration: A sequel to the Wnt-Wnt situation. Hepatol Commun 2, 845–860 . 30027142 10.1002/hep4.1196 PMC6049069

[9] Lanthier N, Spahr L (2019) Resident liver progenitor cells: Proofs of their contribution to human liver regeneration. Clin Res Hepatol Gastroenterol 43, 646–648 . 30878340 10.1016/j.clinre.2019.02.006

[10] Duan JL, Ruan B, Yan XC, Liang L, Song P, Yang ZY, Liu Y, Dou KF, Han H, Wang L (2018) Endothelial Notch activation reshapes the angiocrine of sinusoidal endothelia to aggravate liver fibrosis and blunt regeneration in mice. Hepatology 68, 677–690 . 29420858 10.1002/hep.29834 PMC6099357

[11] Ru YX, Dong SX, Zhao SX, et al. (2019), One cell one niche: hematopoietic microenvironments constructed by bone marrow stromal cells with fibroblastic and histiocytic features. Ultrastruct Pathol 43, 117–125 . 31137995 10.1080/01913123.2019.1620394

[12] Zhai R, Wang Y, Qi L, et al. (2018), Pharmacological mobilization of endogenous bone marrow stem cells promotes liver regeneration after extensive liver resection in rats. Sci Rep 8, 3587 . 29483616 10.1038/s41598-018-21961-2 PMC5827664

[13] Mikael PE, Willard C, Koyee A, et al. (2019), Remodeling of glycosaminoglycans during differentiation of adult human bone mesenchymal stromal cells toward hepatocytes. Stem Cells Dev 28, 278–289 . 30572803 10.1089/scd.2018.0197 PMC6389772

[14] Gupta P, Sata TN, Yadav AK, et al. (2019), TGF-β induces liver fibrosis via miRNA-181a-mediated down regulation of augmenter of liver regeneration in hepatic stellate cells. PLoS One 14, e0214534 . 31166951 10.1371/journal.pone.0214534 PMC6550375

[15] Overi D, Carpino G, Cardinale V, et al. (2018), Contribution of resident stem cells to liver and biliary tree regeneration in human diseases. Int J Mol Sci 19, 2917 . 10.3390/ijms19102917 PMC6213374 30257529

[16] Shi JH, Line PD (2019) Hallmarks of postoperative liver regeneration- an updated insight on the regulatory mechanisms [published online ahead of print, 2019 Nov 29. J Gastroenterol Hepatol, DOI: 10.1111/jgh.14944 . 31782974

[17] Müsch A (2018), From a common progenitor to distinct liver epithelial phenotypes. Curr Opin Cell Biol 54, 18–23 . 29505983 10.1016/j.ceb.2018.02.008 PMC6119654

[18] Li HM (2017), Comprehensive and in-depth study of traditional Chinese medicine regulation of liver regeneration. Chin J Integr Tradit West Med Liver Dis 17, 129–132 .

[19] Li HM (2011), Essence of deficiency syndrome and generate of vitality theory. Chin Arch Tradit Chin Med 29, 2157–2160 .

[20] Qian Y (2018), Jieduan Niwan method for chronic severe hepatitis. Beijing J Tradit Chin Med 27, 85–86 .

[21] Hu JH, Qian Y, Yao NL, Nie G, Wu QK, Li XD, Gou CY, Yang HS, Li XH (2016) Treatment of chronic severe hepatitis B by Jieduan Niwan method. Chin J Integr Tradit West Liver Dis 20, 200–203 .

[22] Wang LF, Li J, Li FY, Zhang XF, Zhang MX, Cao WK, Li Q, Wen XM, Mao DW, Sun KW, Zhou XZ, Tian DY, Guo JC, Yang DG, Li XH, Yang HZ, Wang XB, Zhuo YH, Zhang Q, Li HM, Hu XY, Zhao W, Zhang FC, He LY (2013) A multicentered, randomize-controlled trial of integrative medicine for acute-on-chronic (subacute) liver failure. J Tradit Chin Med 54, 1922–1925 .

[23] Dang ZQ, Yang GH, Ma YJ, Zhao CP, Yu JZ, Xi YH, Wang HX (2017) Synergistic effect of multi-ways chinese medication on routine therapy for hepatitis B virus related acute-on-chronic liver failure. J Tradit Chin Med 53, 2109–2111 .

[24] Liu HM, Wang XB, Hou YX, Gao FY, Sun FX, Jiang YY, Yang ZY, Du HB, Wang XJ, Zhou GQ, Yang YY, Wang RB (2014) Patients with hepatitis B virus related acute-on-chronic liver failure: a randomized controlled clinical study. Chin J Integr Tradit West Med 34, 412–417 . 24812894

[25] Chen YQ, Mao DW, Tang N, Liu LH, Wang N (2015) Efficacy of modified Yinchen Sini Tang in acute-on-chronic liver failure. Chin J Exp Tradit Med Formulae 21, 163–166 .

[26] Huang GY, Mao DW, Long FL, Huang B, Huang ZF, Li DF, Qiu H, Chen C, Zhang RZ, Lv JL, Zeng F (2018) Retention enema with rhubarb decoction in treating hepatic encephalopathy: results from a multi-center clinical trial. Liaoning J Tradit Chin Med 7, 1364–1366 .

[27] Hu XY, Zhang Y, Chen G, Zhong S, Fan XJ (2012) A prospective cohort study on the influence of high doses of herbs for clearing heat and resolving stasis on survival rates in patients with hepatitis B-related acute-on-chronic liver failure. J Chin Integr Med 10, 176–185 . 10.3736/jcim20120208 22313885

[28] Wu XR, Guo WD, Ma XH (2009) Effect of rhubarb on liver injury and liver regeneration in fulminant hepatic failure. Chin J Hepatol 7, 37–38 .

[29] Shi WL, Liu FF, Sun YQ (2015) Thoughts and methods for prevention and treatment of triple factors in the mechanism of hepatitis B-related acute-on-chronic liver failure. J Beijing Univ Tradit Chin Med (Clin Med) 17, 35–37 .

[30] Liu CN, Liu XY, Yang J, Wu GQ, Wang HX (2009) The protective effects of artemisiae capillaris on hepatic damage and its mechanism. Lishizhen Med Mater Med Res 20, 2987–2989 .

[31] Yang M, Wang WD, Zhang LJ, Liu YX, Ma SX (2018) Experimental research on the effects of nanometer Yin Chen mixture to TN F-α and H GF in the serum of rats with fulminant hepatic failure. J Harbin Med Univ 42, 367–370 .

[32] Chen SD, Feng Q, Peng JH, Xu LL, Liu P, Liu C, Hu YY (2009). Research of herba artemisiae scoporiae inhibits the hepatic lipotoxicity. Chin J Chin Mater Med 34, 2373–2378 . 20030092

[33] Sun T, Chen Y (2010) Advances in research on pharmacological effects of Yinchen. Pharm Clin Chin Mater Med 1, 59–61 .

[34] Qi Z, Zheng BZ, Liu JP, Li PY (2016) Advances in studies on biological activities of licorice. Special Wild Economic Animal and Plant Research 38, 71–76.

[35] Shen L, Tao YY, Zhao ZM, Liu HL, Liu CH (2015) Screen active ingredient of liquorice via anti-liver sinusoidal endothelial cells hypoxia injury. Chin J Integr Tradit West Med Liver Dis 25, 227–230 .

[36] Shin T, Kuboki S, Lentsch AB (2018) Roles of nuclear factor-kappaB in postischemic liver. Hepatol Res 38, 429–440 . 10.1111/j.1872-034X.2007.00303.x 18034829

[37] Wu J, Zern MA (1999), NF-kappa B, liposomes and pathogenesis of hepatic injury and fibrosis. Front Biosci 4, D520–D527 . 10369809 10.2741/a447

[38] Qiu HP, Li XL, Yao HP (2016) Study on the mechanism of SNMT on acute liver injury in mice by NF-κB signaling pathway. Chongqing Med 45, 4211–4213 .

[39] Li HM (2009), The scientific connotation of kidney essence. J Tradit Chin Med 50, 1061–1064 .

[40] Li H (2012) EMT/MET imbalance and lost marrow to generate liver. Chin J Integr Tradit West Med Liver Dis 21, 385–389 .

[41] Li H (2015), Research progress in the essence of marrow. J Hubei Univ Chin Med 17, 100–103 .

[42] Li H (2017), Therapeutic principle of “nourishing kidney to produce marrow and generate liver”. Chin Arch Tradit Chin Med 30, 937–940 .

[43] Bihari C, Anand L, Rooge S, Kumar D, Saxena P, Shubham S, Sukriti N, Kumar G, Pamecha V, Sharma S, Rastogi A, Kumar A, Sarin SK (2016) Bone marrow stem cells and their niche components are adversely affected in advanced cirrhosis of the liver. Hepatology 64, 1273–1288 . 27486864 10.1002/hep.28754

[44] Li H, Zhang L, Qiu X, Mei J, Wang P (2011) Study on the mechanism of Zuogui Pill in improving MSG-liver Regeneration-deficiency syndrome of kidney essence and liver blood deficiency in rats. J Hubei Coll Tradit Chin Med 3, 30–33 .

[45] Li HM, Yang M, Mei J, Zhang L, Qiu X (2014) The effects of Zuogui Pill on expression of TGF-α, β and their receptors in rats. Chin J Hepatol 12, 307–308 . 15161515

[46] Li H, Gao X, Yang M, Mei J, Zhang L, Qiu X (2004) Effects of Zuogui Wan on neurocyte apoptosis and downregulation of TGF-beta1 expression in nuclei of acute hypothalamus of monosodium glutamate -liver regeneration rats. World J Gastroenterol 10, 2823–2826 . 15334678 10.3748/wjg.v10.i19.2823 PMC4572110

[47] Li H, Gao X, Zhou M. Zuogui Pill regulates MSG-liver regeneration-related gene expression in rats. Chinese Journal of Basic Medicine in Traditional Chinese Medicine. 2015; 11: 595–598.

[48] Li H, Gao X, Yan X, Ming A, Peng Y, Li J (2005) Study on the effect of Zuogui Pill in promoting bone marrow cells to form hepatic cells. World Chin J Digestol 13, 2818–2822 .

[49] Li H, Gao X, Yan X, Ming A, Peng Y (2016) Study on the molecular mechanism of Zuogui Pill in promoting bone marrow cells to form hepatic cells. J Tradit Chin Med 47, 778–780 .

[50] Li H, Yan X, Luo J, Li J, Gao X, Ming A, Peng Y (2017) Effect of Zuogui Pill medicated serum on the differentiation of bone marrow mesenchymal stem cells into hepatocytes. J Clin Rehab Tissue Eng Res 11, 5465–5468 .

[51] Li H, Gui W, Li J, Gao X, Yan X, Cheng Y (2008) Effect of Zuogui Pill on the liver regeneration related gene signal pathway in female mice with female mice bone marrow transplant. J Clin Rehab Tissue Eng Res 12, 6069–6073 .

[52] Gao X, Li H, Yan X (2010) Effects of Zuogui Pill on wnt signal pathway in female mice with male mice bone marrow transplant. Chin J Integr Tradit West Med Liver Dis 20, 29–31 .

[53] Li H, Gao X, Yan X (2015) Studies of Zuogui Pill medicated serum pharmacology based on co-culture system of bone marrow stem cells and hepatocytes. J Clin Rehab Tissue Eng Res 14, 3527–3532 .

[54] Song H, Li H, Li L, Gao X, Zhao B, Zhang J, Wu Y, Yan X, Xiao L (2013) Effects of Diwuyanggan capsule on liver regeneration of rat with deficiency of kidney essence and liver blood. Chin J Integr Tradit West Med Liver Dis 23, 90–92 .

[55] Li H (2013), The basic and clinical application of “tonifying kidney to generate marrow and liver” in the treatment of liver disease. World Sci Technol/Modern Tradit Chin Med Mater 15, 1425–1428 .

[56] Li H (2018), Introduction to regenerative traditional Chinese medicine. Chin Arch Tradit Chin Med 26, 2309–2312 .

[57] Wu X, Huang B, Feng B (2012) Advances in molecular mechanism of liver regeneration. Chin Med Pharm 2, 64–66 .

[58] Zhang AM (2012) Clinical efficacy and mechanism in the treatment of chronic severe hepatitis by heat-clearing, detoxifying and dampness-removing methods. Jiangxi J Tradit Chin Med 43, 23–27 .

[59] Gao Y, Chen GM, Liang ZY, Xie YQ (2013) Advances in research on the effects of cytokines on liver fibrosis. Hainan Med J 24, 111–113 .

[60] Li Y, Ma SP (2015), A TCM study on correlation between TNF-α, IL-6 and hepatitis B. Clin J Chin Med 7, 146–148 .

[61] Parola M, Pinzani M (2019) Liver fibrosis: Pathophysiology, pathogenetic targets and clinical issues. Mol Aspects Med 65, 37–55 . 30213667 10.1016/j.mam.2018.09.002

[62] Friedman SL (2008), Hepatic fibrosis-overview. Toxicology 254, 120–129 . 18662740 10.1016/j.tox.2008.06.013

[63] Kordes C, Sawitza I, Müller-Marbach A, Ale-Agha N, Keitel V, Klonowski-Stumpe H, Häussinger D (2007) CD133+ hepatic stellate cells are progenitor cells. Biochem Biophys Res Commun 352, 410–417 . 17118341 10.1016/j.bbrc.2006.11.029

[64] Li HM (2015) Prevention and treatment of liver fibrosis by regulating liver regeneration. J Clin Hepatol 31, 992–994 .

[65] Shen X, Cheng S, Peng Y, Song H, Li H (2014) Attenuation of early liver fibrosis by herbal compound “Diwu Yanggan” through modulating the balance between epithelial-to-mesenchymal transition and mesenchymal-to-epithelial transition. BMC Complement Altern Med 14, 418 . 25345787 10.1186/1472-6882-14-418 PMC4218991

[66] Cheng SS, Shen X, Peng Y, Li HM (2015) Effect of Diwu Yanggan Capsule on EMT/MET of liver tissue in rats with hepatic fibrosis. Lishizhen Med Mater Med Res 26, 826–829 .

[67] Shen X, Peng Y, Cheng SS, Li HM (2015) Regulation effect of Diwu Yanggan Capsule on the Hedgehog signal pathway in the liver tissues of fibrotic rats. Chin J Tradit Chin Med Pharm 30, 2955–2957 .

[68] Dusabineza AC, Van Hul NK, Abarca-Quinones J, Starkel P, Najimi M, Leclercq IA (2012) Participation of liver progenitor cells in liver regeneration: lack of evidence in the AAF/PH rat model. Lab Investig 92, 72–81 . 21912377 10.1038/labinvest.2011.136

[69] Dezső K, Papp V, Bugyik E, Hegyesi H, Sáfrány G, Bödör C, Nagy P, Paku S (2012), Structural analysis of oval-cell-mediated liver regeneration in rats. Hepatology 56, 1457–1467 . 22419534 10.1002/hep.25713

[70] Zhang W, Chen XP, Zhang WG, Zhang F, Xiang S, Dong HH, Zhang L (2009) Hepatic non-parenchymal cells and extracellular matrix participate in oval cell-mediated liver regeneration. World J Gastroenterol 15, 552–560 . 19195056 10.3748/wjg.15.552 PMC2653345

[71] Zhao BB, Li HM, Gao X, Ye ZH, Cheng SS (2015) The herbal compound “diwu yanggan” modulates liver regeneration by affecting the hepatic stem cell microenvironment in 2-acetylaminoflfl uorene/partial hepatectomy rats. Evid Based Complement Alternat Med 2015, 468303 . 25628749 10.1155/2015/468303 PMC4299675

[72] Li H, Zhang L (2017) Liver regeneration microenvironment of hepatocellular carcinoma for prevention and therapy. Oncotarget 8, 1805–1813 . 27655683 10.18632/oncotarget.12101 PMC5352100

[73] Li HM (2016), Microcirculation of liver cancer, microenvironment of liver regeneration, and the strategy of Chinese medicine. Chin J Integr Med 22, 163–167 . 26919996 10.1007/s11655-016-2460-y

[74] Li HM (2015), Construction and application of tertiary prevention program for liver cancer based on “tonifying the kidney to promote liver regeneration and repair by affecting stem cells and their microenvironment”. Chin J Integr Tradit West Med Liver Dis 25, 369–372 .

